# Production and biochemical characterization of *Pleurotus ostreatus* NRC 620 laccase and evaluation of its efficacy in apple juice clarification

**DOI:** 10.1038/s41598-025-04992-4

**Published:** 2025-06-20

**Authors:** Ali M. Elshafei, Maysa A. Elsayed, Gamil E. Ibrahim, Nayra S. Mehanna, Mohamed M. Hassan, Abdelmageed M. Othman

**Affiliations:** 1 Microbial Chemistry Department, Biotechnology Research Institute, National Research Centre, 33 El Bohouth St., Dokki, Giza, 12622 Egypt; 2 Chemistry of Flavor and Aroma Department, Food Industries and Nutrition Research Institute, National Research Centre, 33 El Bohouth St., Dokki, Giza, 12622 Egypt; 3 Dairy Sciences Department, Food Industries and Nutrition Research Institute, National Research Centre, 33 El Bohouth St., Dokki, Giza, 12622 Egypt; 4 Faculty of Biotechnology, German International University, Regional Ring Road, East Cairo, New Administrative Capital, Cairo, Egypt

**Keywords:** Biochemical characterization, *Pleurotus ostreatus*, Laccase, Juice clarification, Biochemistry, Biological techniques, Biotechnology

## Abstract

The highest activity of the *Pleurotus ostreatus* NRC620 laccase enzyme occurred in the broth of mushroom growth after 25 days of incubation at 28 °C and static conditions. The optimum pH and temperature of the enzyme activity were revealed at pH 3.0, and 70 °C, respectively, and retained 68.33 and 59.61% of its activity after incubation at 40 and 50 °C for 2 h, respectively. The enzyme retained 100% activity after 2 h of incubation in citrate–phosphate buffer (pH 7.0). The addition of MgSO_4_ and CuSO_4_ with concentrations of 10 mM caused about 21% and 35% increase in enzyme activity, while NaCl, MnCl_2_, KCl, and CaCl_2_ inhibited the enzyme. The values of the kinetic parameters (K_*m*_ and V_*max*_) of the *Pleurotus ostreatus* NRC 620 laccase were 1.99 mM and 16,217 µmol Min^−1^ L^−1^ respectively, using ABTS as a substrate. The apple juice enzyme-treated sample showed a significant reduction in pH, as well as viscosity, after enzyme treatment along with storage time. Apple juice treatment with laccase exhibited slight degradation in total phenolic; however, this observation was not found in antioxidant activity.

## Introduction

In recent years, researchers have focused their efforts on using environmentally friendly biotechnology in the food business. Laccase is one of the most useful enzymes in the food business, including fruit juice processing, baking, wine stability, and improving food sensory qualities^1^. Many types of higher plants and microorganisms secrete laccase^2^, also laccase is produced by fungi from the Deuteromycetes, Ascomycetes, and Basidiomycetes^3^. By reducing molecular oxygen to water via a system of three different kinds of copper atoms, laccase (EC1.10.3.2), a blue oxidase, may oxidize a variety of phenolic compounds as well as aromatic amines. When processing fruit and vegetable juice, browning, whether enzymatic or non-enzymatic, is one of the most important issues^4^. Since these substances negatively impact the color, flavor, and scent of fruit juice, they have to be removed^5^.

Of all the fruits, apples are the most significant and consumed in the world and in the EU. In 2019, they were grown in the third-largest quantity worldwide, with over 87 million tons^6^. Numerous phenolic chemicals, including flavonoids and phenolic acids like caffeic and chlorogenic acids, are found in apples^7^. Since apple juice is frequently utilized as clear juice, phenolic components are lost during filtration to the tune of around 50–90%^8^. Foods with minimum processing, such as cloudy apple juices with high polyphenol contents, are preferred by customers these days. However, because of their high phenolic component contents, they are particularly vulnerable to color fading and browning^9^. A number of techniques, including thermal technology with pasteurization at 60–90 °C, were employed to reduce or stop apple juice browning^10^. According to Sauceda-Gálvez^11^, using heat treatments damages volatile chemicals and unfavorable sensory qualities. Alternative techniques for thermal technologies include CO_2_ supercritical, UV light, ultrasounds, high hydrostatic pressure, or high-pressure homogenization^12^. The effectiveness of these techniques and the yield of suitable juice products are dependent on the parameters employed and the features of the product. Due to their expensive cost, detrimental effects on some food qualities, or inadequate enzyme inactivation, their widespread use has been restricted^13,14^.

The fruit juices might be stabilized and clarified by using the laccase enzyme^15^. Gökmen et al.^16^ suggested using laccase for juice clarification because it effectively removes phenol by converting it into polymers or oligomers that are easily removed by any ultrafiltration membrane, resulting in apple juice that is stable in color and clarity for up to six weeks at 50 °C. Purified *Trametes hirsuta* laccase was immobilized on alumina oxide beads and utilized to selectively remove off-flavor compounds from apple juice that were brought on by microbial contamination^17^.

About 80–90% of the volatile components in apple juice are esters and aldehydes, which are responsible for its scent^18^. The *Trametes versicolor* laccase was utilized to clarify apple juice after being immobilized on inexpensive support made of natural fibers from green coconut husk^19^. Investigations have been conducted on the stabilization of apple juice (color and turbidity) using either an enzyme-free or immobilized approach alone or in conjunction with ultrafiltration^5,19^. On the other hand, nothing is known about how fungal laccase affects the physicochemical characteristics of apple juice when it is being stored. Thus, this study is an experiment to monitor how apple juice’s physicochemical characteristics, phenolic chemicals, and antioxidant activities change after being treated with fungal laccase and refrigerated for two weeks. Laccases’ capacity to oxidize phenolic chemicals makes them useful in a wide range of industrial processes, including juice clarity. The laccase from *Pleurotus ostreatus* NRC 620 is examined in this work, with an emphasis on its ideal activity conditions and efficiency in juice clearing. While *P. ostreatus* NRC 620 has not been thoroughly investigated, enzymes from several fungal sources, like *Trametes versicolor* and *Ganoderma lucidum*, have been previously researched. The purpose of this study is to assess the enzyme’s potential in the food business and to emphasize its special qualities, especially its ideal pH and temperature.

## Materials and methods

### Chemicals

2, 2´-azino-bis (3- ethylbenzothiazoline-6-sulphonic acid) (ABTS) from Sigma-Aldrich (Canada) was acquired. Every other substance was analytical grade.

### Microorganism and enzyme production

The National Research Center’s culture collection yielded the well-known *Pleurotus ostreatus* NRC620 mushroom. The strain was subcultured and kept at 4 °C on potato dextrose agar slants. The inoculum was prepared on potato dextrose agar plates using ten-day-old fully developed slants at 28 °C. Following 10 days of incubation, three 12-mm disks of fungal mycelia were removed from the agar plates using a sterile metal cork borer and placed in 250 mL cotton-plugged Erlenmeyer flasks with 50 mL of the sterilized media (pH 5.0) that Othman et al.^20^ had previously described. For eighteen days, the culture was maintained in a static thermo-controlled incubator at 28 °C. Following that, Whatman No. 1 filter paper was used to filter the culture, and the source of the enzyme was the acquired supernatant.

### Enzyme assays and protein estimation

Laccase activity was measured using ABTS as the substrate. The reaction mixture (2 mL) included 500 μL of 0.3 mM ABTS mixed in 0.1 M sodium citrate buffer pH 4.5, as well as the necessary amounts of diluted enzyme sample with distilled water^21,22^. Considering that laccase activity oxidizes ABTS at ambient temperature (28 °C ± 2). The Agilent Carry-100 UV–Vis Spectrophotometer, made in Germany, might therefore be used to detect ABTS oxidation by measuring the absorbance increase at 420 nm (ɛ420 = 36,000 cm^−1^ M^−1^). One μ mole of ABTS may be oxidized each minute with one unit of laccase activity, which is the quantity of enzyme needed. Using bovine serum albumin as a reference protein, Bradford’s technique was used to quantify the protein concentration^23,24^.

### Optimization of incubation time for maximum laccase production

After the *Pleurotus ostreatus* NRC 620 strain produced the enzyme, the activity of the enzyme was measured during a 25-day period at various incubation intervals under static circumstances at 28 °C.

### The effect of temperature on laccase activity and stability

To investigate the effect of temperature on laccase activity, a range of temperatures from 20 to 90 °C was employed. Prior to introducing the enzyme and initiating the reaction, the buffer (0.1 M sodium citrate, pH 4.5) and substrate (ABTS) were combined and incubated for five minutes at different temperatures. The thermal stability of the enzyme was assessed by incubating it for two hours at various temperatures of 40, 50, 60, and 70 °C in a 0.05 M sodium phosphate buffer (pH 7.0). The substrate, ABTS, was then used to assess residual activity.

### Effect of pH on the activity and stability of laccases

The influence of pH values on laccase activity was assessed by using ABTS as a substrate in a buffer containing 0.1 M citrate–phosphate buffer, with pH values ranging from 2.5 to 7.0. For two hours, the enzyme’s pH stability was assessed by soaking the enzyme solution in 0.1 M citrate and Tris buffers (pH 3, 4, 6, and 7) at 40 °C. After incubation, the residual activity using ABTS as a substrate was computed.

### Effects of metal ions on laccase activity

Laccase was incubated for 10 min in a sodium phosphate buffer (0.05 M, pH 7.0) that included various metal ions, such as Mg^2+^, Cu^2+^, Co^2+^, Ca^2+^, Zn^2+^, K^+^, Na^+^, and Mn^2+^, at concentrations of 2.5 and 10 mM. After that, the reaction was initiated by adding the substrate (ABTS), and then the relative activity was assessed.

### Kinetic parameters

The oxidation of ABTS by laccase at several doses (0.025–3 mM) was measured at pH 4.5 to determine the kinetic parameters (*V*_*max*_ and *K*_*m*_). The Michaelis–Menten equation’s kinetic constants were calculated using the Lineweaver–Burk plots of the reciprocal of reaction velocities and substrate concentrations^25,26^. Using the Lineweaver–Burk plot, the kinetic constants were obtained using GraphPad Prism version 6.01.

### Preparation of apple juice and its treatment by laccase

After properly washing the apples with tap water, they were sliced in half and juiced using a Multipress automated Braun MP80 apple juice extractor from Germany. After that, a 4-layer cheesecloth is used to filter the prepared juice. While the control sample had no enzyme addition, the freshly prepared apple juice was treated with 2.0% laccase (as the most effective concentration among tested concentrations) and then incubated at 4 °C for two weeks.

### Physicochemical properties of treated and untreated apple juice

#### pH values and titratable acidity (TA)

The measurements of titratable acidity (TA) and pH values were made in accordance with Boulton et al.^27^. Using a digital pH meter (JENWAY 3510 pH meter), the pH values of every sample were determined. Using the following formula, malic acid was used to compute titratable acidity (TA).

$$TA\% = \frac{V \times C \times K}{W} \times 100$$

where V and C stand for the amount of sodium hydroxide solution (ml) and concentration (0.1 mol/L) used in the titration procedure, respectively. K is the malic acid conversion coefficient, which is 0.067, and W is the weight of apple juice (g).

#### Total soluble solids (TSS)

All juice samples were measured for TSS using a Pocket Refractometer PAL-1 (ATAGO, Tokyo, Japan)^28^. After washing the optical lens with deionized water between measurements, each of the apple juice samples under study was tested three times. A value was produced for each of the prepared samples by averaging the readings for the triplicates. For each sample of apple juice, the mean ± SD was calculated by averaging those results.

#### Rheological measurements

The rotating type RV (Rheotest 2-Germany) was used to assess the viscoelasticity of the apple juice samples under study. The samples under investigation were placed within the viscometer’s “S2” cylinder. The entire apparent viscosity was represented by the slope of the relationship between shear stress and shear rate, which was derived from the produced shear stresses at various shear rates ranging from 1.00 to 437.4 Sec^−1^ and their related curves. Using the following formula, the apparent viscosity was determined:

$$Apparent\;viscosity\;\eta = \tau /\gamma \times 100$$

where η is the apparent viscosity in cp, τ is the shear stress (dyn/cm^2^), γ is the shear rate (sec^−1^), and (τ) was computed using the following formula using the torque value (α) and the cylinder (Z): τ = Z. α.

#### Browning index (BI)

The Meydav et al.^29^ technique was used to measure the browning index. A 10 ml sample of juice was centrifuged for 10 min at 2750 xg. Five milliliters of 95 percent ethyl alcohol were mixed with five milliliters of juice supernatant. At 420 nm, the mixture’s absorbance was measured with a UV–Vis Shimadzu Spectrophotometer (UV-1601 PC).

### Phytochemical and antioxidant activity analysis

#### Total phenol content (TPC)

The Folin–Ciocalteau technique was used to colorimetrically estimate TPC in accordance with the approach described by Boulton et al.^27^. A gallic acid standard curve with a range of 0 to 500 mg/L was created (r^2^ = 0.997). Gallic acid equivalent (mg GAE/mL) was used to estimate the findings.

#### Antioxidant activity measurements

- Ferric reducing antioxidant power (FRAP) assay

After adding 125 μL of distilled water and 2850 μL of FRAP solution to 25 μL of apple juice, the mixture was kept in the dark for half an hour^30^. After that, the UV–Vis Shimadzu Spectrophotometer (UV-1601 PC) was used to detect the absorbance at 593 nm. To make the FRAP reagent, 300 mM acetate buffer (pH 3.6), 20 mM ferric chloride, and 10 mM 2,4,6-Tri(2-pyridyl)-s-triazine (TPTZ) in 40 mM HCl were mixed in a 10:1:1 ratio. Trolox was established as a standard curve (R^2^ = 0.999), and the findings were expressed as µM Trolox/mL.

b. DPPH assay

Using the DPPH technique, the antioxidant activity of the treated and untreated juices was also examined to determine their capacity to scavenge the DPPH^·^ free radical^31^. Ten microliters of juice were mixed with one milliliter of DPPH^·^ (100 μM) methanolic solution. After 30 min in the dark, the mixture was tested for absorbance at 517 nm using a UV–Vis Shimadzu Spectrophotometer (UV-1601 PC). Following the acquisition of the matching calibration curve (R^2^ = 0.990), the results were represented as Trolox equivalent (µM Trolox/mL).

## Results and discussion

### Effect of incubation time on the production of *Pleurotus ostreatus* NRC 620 laccase

**Fig. 1:**
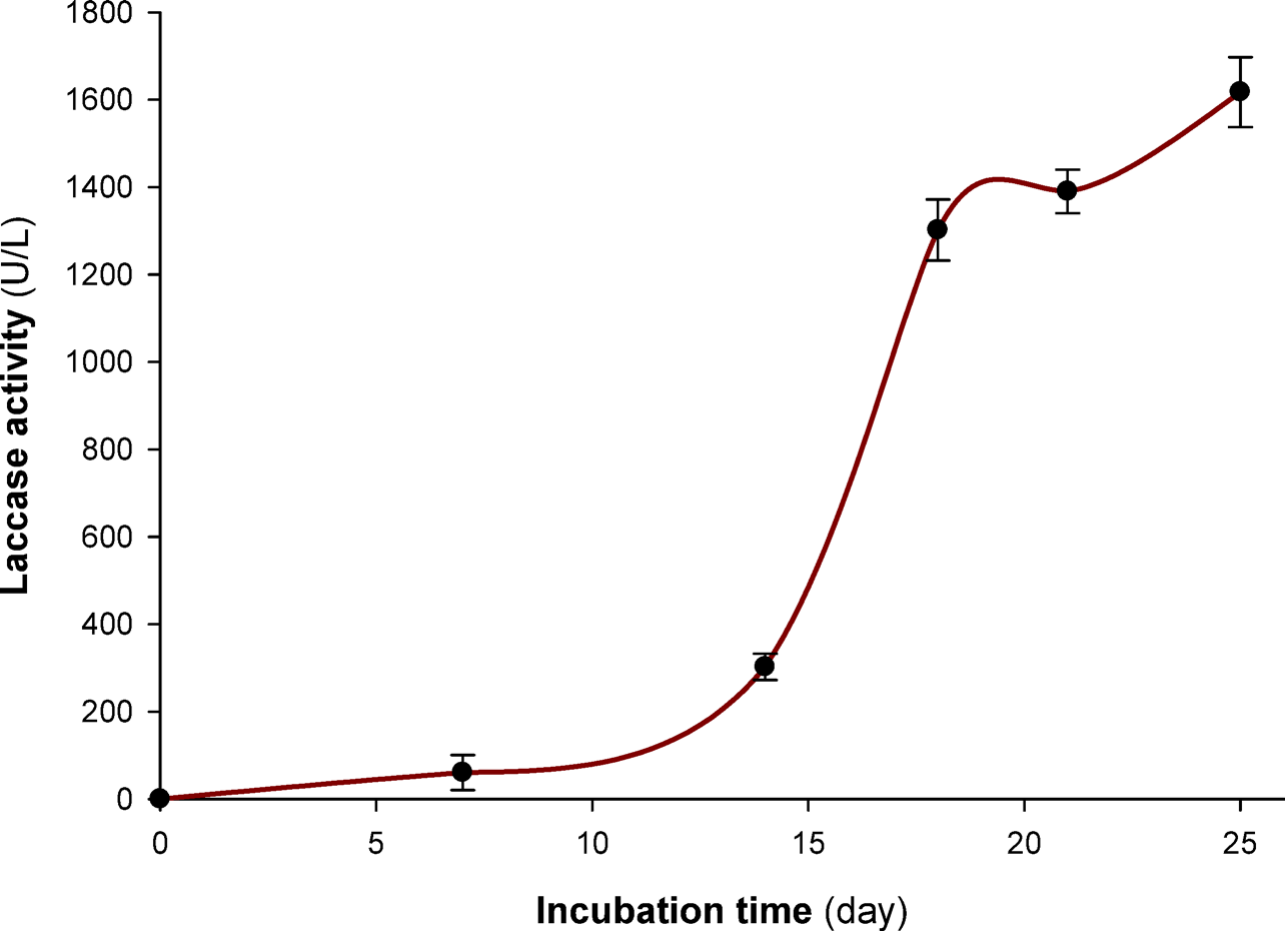
Effect of incubation time on the production of *Pleurotus ostreatus* NRC 620 laccase. Three (12-mm) disks of fungal mycelia were used to inoculate 50 mL sterile media and then incubated at 28 °C for different incubation periods.

The data obtained indicated that *Pleurotus ostreatus* NRC 620 generated the greatest rate of laccase by the conclusion of the eighteenth day of fermentation, with an activity of 1302 U/L, in an attempt to determine the ideal incubation period for laccase production (Fig. 1). While the amount of enzyme generated increases with longer incubation times, the rate of growth is not proportionate to the period of incubation; after 21 days, the activity only rose by 90 U/L (1390 U/L). Because of this, 18 days was chosen as the ideal incubation period to balance the amount of product produced with the financial benefits of longer incubation.

Consistent with other research, our findings indicate that the ideal incubation period for fungi to produce the highest amount of laccase secretion may range from 7 to 36 days^32^. *Trametes polyzona* WRF03 produced the most laccase at the conclusion of the ninth day of fermentation, with a specific activity of 1637 U/mg protein, according to Ezike et al.^33^. Furthermore, Othman et al.^34^ found that during the fifth day of incubation, *Trichoderma harzianum* S7113 was secreting a significant amount of laccase enzyme. The laccase enzyme’s production rate began to progressively decrease after reaching its highest peak of activity on the fourteenth day^34^. While enzyme secretion may occur during the main growth phase, it typically occurs at its highest level during the idiophase and is triggered by the depletion of carbon or nitrogen supplies^34,35^.

### Effect of reaction temperature on the activity of *Pleurotus ostreatus* NRC 620 laccase

**Fig. 2:**
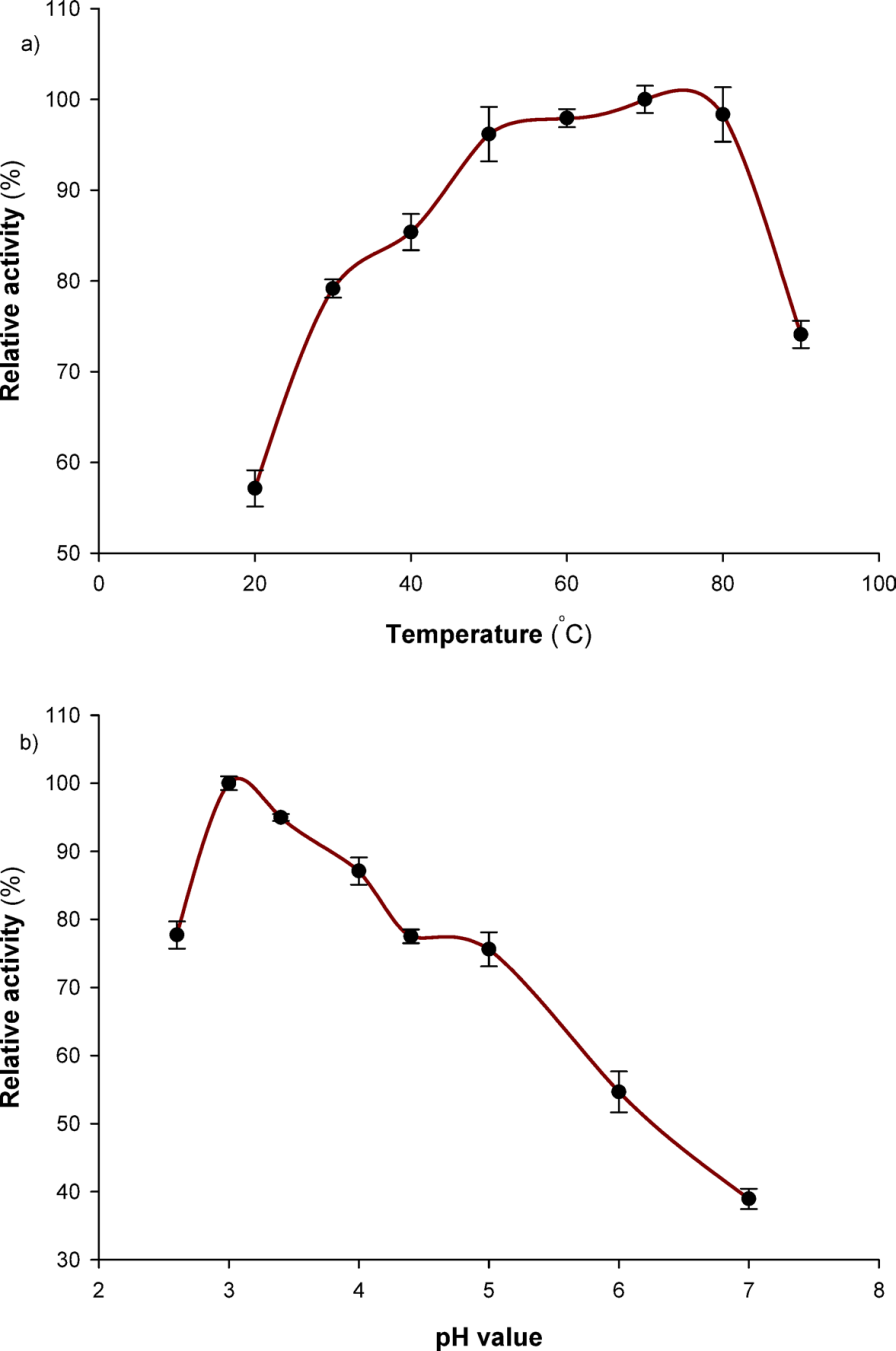
Effect of reaction temperature (**a**) and pH (**b**) on the activity of *Pleurotus ostreatus* NRC 620 laccase. A range of temperatures from 20 to 90 °C was employed through prior mixture temperature maintaining (5 min.) at required different temperatures before introducing the enzyme and initiating the reaction. The influence of pH values on laccase activity was assessed by using ABTS as a substrate in a buffer containing 0.1 M citrate–phosphate buffer, with pH values ranging from 2.5 to 7.0.

Although it was most active throughout a wide temperature range from 50 °C to 80 °C to almost reach its peak activity (69–98%), the *Pleurotus ostreatus* NRC 620 laccase enzyme showed its maximum activity at 70 °C (Fig. 2a). Moving away from this temperature focus zone decreased the enzyme activity both before and after these levels. These findings suggest that the enzyme under study is active at high temperatures, which might be because high temperatures enhance the kinetic energy of reactions.

According to Ezike et al.^33^, *T. polyzona* WRF03 laccase exhibits an optimal temperature of 55 °C, which is also the same as that seen for Ganoderma lucidum laccase^36^ and similar for laccase from *Trametes polyzona* KU-RNW027 at 50 °C^37^. The ideal temperature range for laccase, like that of other ligninolytic enzyme systems, is between 50 and 70 °C, according to Baldrian^38^.

### Effect of pH on *Pleurotus ostreatus* NRC 620 laccase activity

Results obtained indicated that the enzyme was most active at pH 3.0 and reached 94% at pH 3.5. Nonetheless, it demonstrated action throughout a broad pH range of 2.5 to 7.0 (Fig. 2b). Also, compared to the neutral or alkaline zone, it was more active in the acidic zone. While the activity ratio was able to preserve at least 77% of its activity between pH values of 2.5 and 4.5, it only attained about 38% of its activity at pH 7.0. The laccase from *Trametes polyzona* WRF03 has a pH optimum of 4.5^33^, which is the same as the laccase produced from *Trametes polyzona* KU-RNW027^37^, *Trichoderma harzanium*^39^, *Pleurotus sp.*^40^ and *Trametes hirsuta*^41^. However, according to Chairin et al.^42^, laccase from *Trametes polyzona* WR710-1 has a pH optimum of 2.2, whereas *Trametes versicolor* IBL-04 laccase gave an ideal pH of 5.0^43^. The binding of a hydroxide anion (a laccase inhibitor) to the T_2_/T_3_ coppers of laccase may be the cause of the reduction in laccase activity at neutral or alkaline pH. This might disrupt the internal electron transfer from T_1_ to T_2_/T_3_ centers, hence limiting the enzyme activity^23,44^.

### Thermal stability of *Pleurotus ostreatus* NRC 620 laccase

**Fig. 3:**
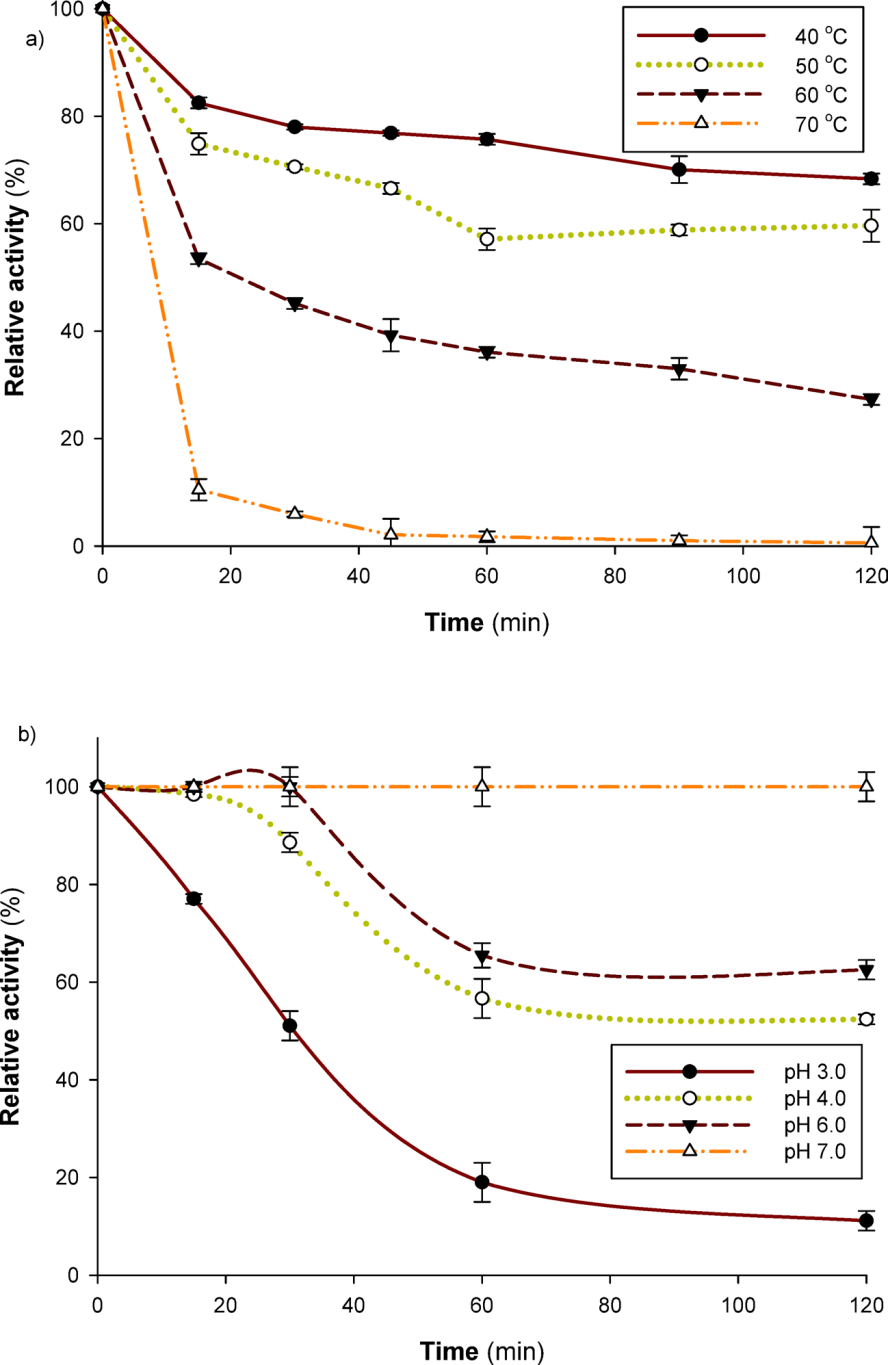
Thermal (**a**) and pH (**b**) stability of *Pleurotus ostreatus* NRC 620 laccase. The thermal stability of the enzyme was assessed by incubating it for two hours at various temperatures of 40, 50, 60, and 70 °C in a 0.05 M sodium phosphate buffer (pH 7.0). For two hours, the enzyme’s pH stability was assessed by incubating the enzyme solution in 0.1 M citrate and Tris buffers (pH 3, 4, 6, and 7) at 40 °C. After incubation, the residual activity using ABTS as a substrate was computed.

It was discovered by incubating the enzyme at various temperatures that the length and temperature of the incubation had an impact on the stability of the enzyme. It is noteworthy that *Pleurotus ostreatus* NRC 620 laccase exhibited greater stability at 40 and 50 °C, retaining 68.33 and 59.61% of its initial activity, respectively, after 120 min (Fig. 3a). In contrast, *Trametes polyzona* WRF03 laccase retained 64.38 and 42.92% of its activity under the same conditions (40 and 50 °C, 120 min)^33^. Conversely, increasing the exposure time and temperature reduced the stability of the *Pleurotus ostreatus* NRC 620 laccase enzyme to 39.24 and 1.72% after 60 min at 60 and 70 °C, respectively (Fig. 3a). Consistent with the results, *Trametes polyzona* WRF03 laccase exhibited greater stability at 40 and 50 °C throughout the heat treatment process^33^. Similarly, Lueangjaroenkit et al.^37^ and Chairin et al.^42^ stated stability for *Trametes polyzona* KURNW027 and *Trametes polyzona* WR710-1 laccase up to 50 °C for one hour, respectively. A useful biocatalyst for several biotechnological applications, laccase should exhibit stability and can function throughout a wide temperature range.

### pH stability of *Pleurotus ostreatus* NRC 620 laccase

To ascertain the ideal circumstances needed for enzyme application and storage, the impact of pH on laccase stability was investigated. Exposure to varying pH levels has a significant impact on the stability of protein structure, which in turn influences the stability of enzyme molecules and, consequently, their activity. The results showed that the enzyme was less stable in acidic conditions and more stable at higher pH levels (in neutral and alkaline zones). At pH levels of 7.0, 6.0, 4.0, and 3.0, the enzyme retained about 100%, 62.54%, 52.39%, and 11.14% after 120 min, respectively (Fig. 3b). *Trametes polyzona* WRF03 laccase was more stable at neutral pH values (5.5–6.5) and less stable at acidic pH values (below 4.0). After 120 min, the enzyme retained around 82%, 100%, and 93% at pH values of 5.5, 6.0, and 6.5, respectively^33^. Chairin et al.^42^ stated that laccase from *Trametes polyzona* WR710-1 was shown to be stable between pH 6.0 and 7.0, although Sayyed et al.^45^ shown that laccase is more stable at neutral pH. Nevertheless, laccase from *Cerrena unicolor* was shown to be stable at alkaline levels (pH 9.0)^46^. Over a wide pH range, the laccase under study exhibited a high degree of stability. For industrial applications, this might be a crucial feature.

### Metal ions

**Fig. 4:**
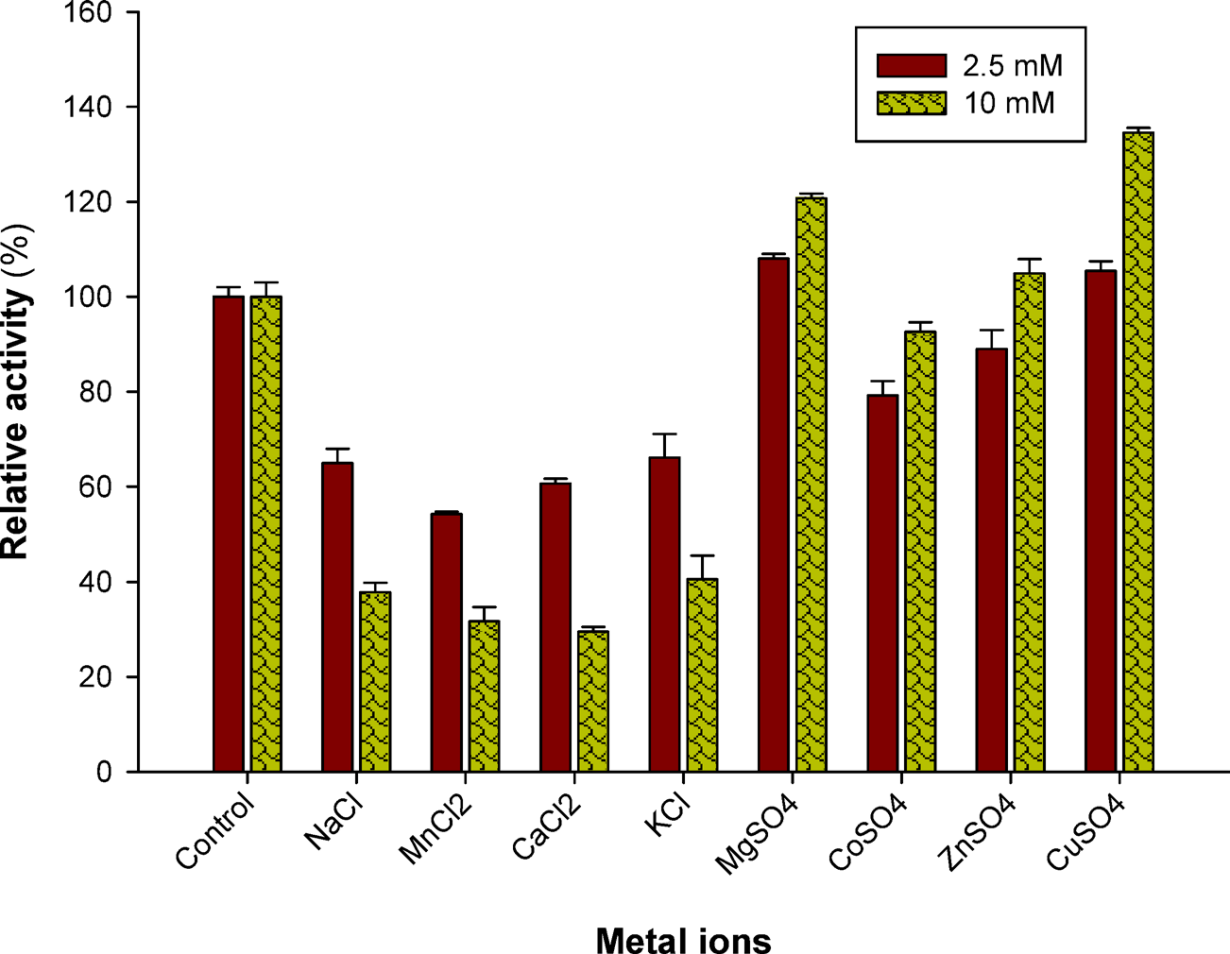
Metal ions effect on *Pleurotus ostreatus* NRC 620 laccase activity. Laccase was incubated for 10 min in a sodium phosphate buffer (0.05 M, pH 7.0) that included various metal ions at concentrations of 2.5 and 10 mM. After that, the reaction was initiated by adding the substrate (ABTS), and then the relative activity was assessed.

Since some metal ions have both stimulatory and inhibitory effects on enzyme activity, their impact on enzyme activity is taken into account in industrial applications. This is crucial because metal ions are prevalent environmental contaminants that might impact extracellular enzyme stability and synthesis^47^. To find out how several metal ions affected *Pleurotus ostreatus* NRC 620 laccase, tests were conducted. According to Fig. 4, depending on the kind of metal utilized, an increase in metal ion concentration from 2.5 mM to 10 mM has a negative impact on enzyme function. The enzyme activity may be stimulated and activated, as in the cases of Mg^2+^, Co^2+^, Zn^2+^, and Cu^2+^, or it may be inhibited, as in the cases of Na^+^, Mn^2+^, Ca^2+^, and K^+^. The greatest activators of *Pleurotus ostreatus* NRC 620 laccase activity were Cu^2+^ and Mg^2+^ at a concentration of 10 mM, which provided activation rates of around 34% and 20%, respectively. However, the strongest inhibitor of laccase was Ca^2+^ at a concentration of 10 mM, which reduced enzyme activity by around 60%.

This study’s findings are consistent with those of other authors, who found that Mg^2+^ and Cu^2+^ enhanced *Trametes polyzona* WRF03 activity^33^. Castaño et al.^48^ found that laccase from *Xylaria sp*. was somewhat stimulated by copper ions (Cu^2+^). In addition, Forootanfar et al.^49^ and Si et al.^50^ revealed comparable findings for the laccases derived from *Paraconiothyrium variabile* and *Trametes pubescens* respectively. The type 2 (T_2_) copper binding sites of the enzyme may be saturated with Cu^2+^ at this concentration, which would explain the stimulation of laccase activity at greater Cu^2+^^39^. Because white rot fungal laccases are oxidases that contain several copper atoms, cupric ions can have a variety of impacts on laccase activity, whether they be positive, negative, or neutral^51^. In contrast, Zhou et al.^52^ reported that Cu^2+^ inhibits *Odontotermes formosanus* laccase. However, laccases from *Cerrena sp*. HYB07^53^, and *Clitocybe maxima*^54^ were unaffected by cupric ions.

### Kinetic parameters

**Fig. 5:**
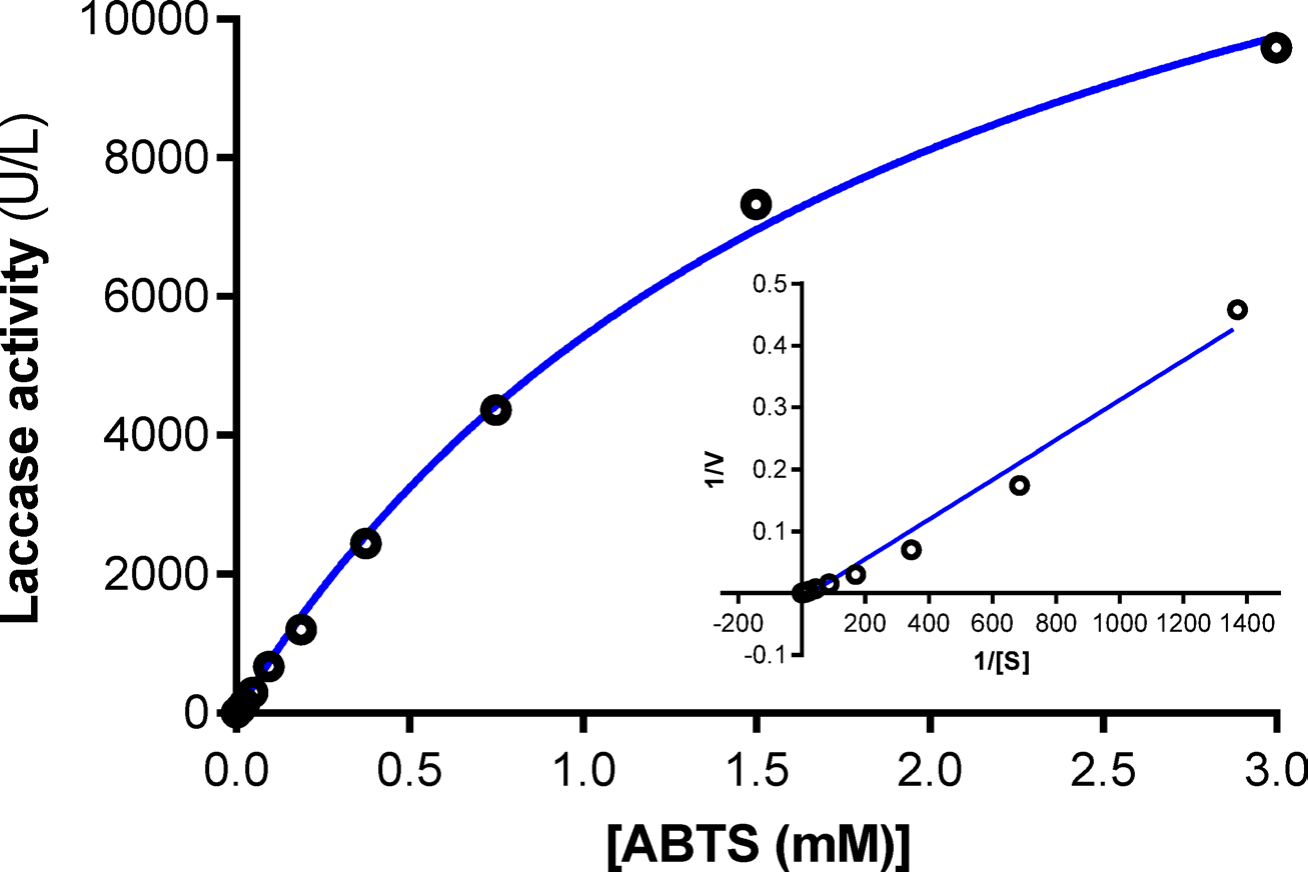
Effect of ABTS concentration on *Pleurotus ostreatus* NRC 620 laccase activity and Lineweaver-Bulk plot of the reciprocal of initial velocities and ABTS concentrations. The oxidation of ABTS by laccase at several doses (0.025–3.0 mM) was measured at pH 4.5 to determine the kinetic parameters (V_max_ and K_m_). The Michaelis–Menten equation’s kinetic constants were calculated using the Lineweaver–Burk plots of the reciprocal of reaction velocities and substrate concentrations. Using the Lineweaver–Burk plot, the kinetic constants were obtained using GraphPad Prism version 6.01.

A substrate’s specificity value is shown by its kinetic parameters (*K*_*m*_ and *V*_*max*_); the more a substrate can bind to an enzyme, the lower its Km value and the higher its substrate specificity^3,21,55^. The *Pleurotus ostreatus* NRC 620 laccase’s kinetic parameters (*K*_*m*_ and *V*_*max*_) were determined using the Lineweaver–Burk plot using GraphPad Prism 6.0 software (Fig. 5). The results were 1.99 mM and 16217 µmol min^−1^ L^−1^ using ABTS as the substrate. Elsayed et al.^21^ claimed that the *K*_*m*_ values of 0.1 and 0.064 mM for ABTS oxidation indicated that the Lac A and Lac B isoenzymes had a high affinity for ABTS. Additionally, the *V*_*max*_ values were 0.182 μmol min^−1^ and 0.603 μmol min^−1^. The obtained *K*_*m*_ value was less than the 8.66 mM value of *Trametes polyzona* WRF03; moreover, their study’s *V*_*max*_ value (1429 mmol/min) using ABTS as the substrate was likewise lower^33^. Likewise, *Lentinus squarrosulus* MR13 and *Trametes sp.* AH28-2 laccases exhibited *K*_*m*_ values of 0.0714 mM and 0.025 mM, as well as *V*_*max*_ values of 0.0091 mM min^−1^ and 0.67 mM min^−1^ mg^−1^, in relation to ABTS^56,57^.

### Impact of laccase treatment on physicochemical characteristics of apple juice

Traditional clarification enzymes such as pectinases hydrolyze pectic substances, reducing viscosity and turbidity. They are highly effective in breaking down structural polysaccharides and are often used in combination with other enzymes like cellulases and hemicellulases to improve yield and clarity. However, pectinases do not specifically target phenolic compounds, which are major contributors to haze formation and oxidative browning — particularly in juices like apple and grape^58^. In contrast, laccase catalyzes the oxidation of phenolic compounds, polymerizing them into larger, insoluble forms that can be removed via sedimentation or filtration. This mechanism not only improves clarity but also enhances shelf-life stability by reducing the potential for phenol-related oxidative browning. Moreover, laccase-based clarification can operate under mild processing conditions (pH 3.5–5.5 and 25–40 °C), making it suitable for delicate fruit juices without affecting nutritional or sensory properties^59^. Studies have shown that while pectinase treatment may clarify juice within 1–2 h, laccase treatment typically requires longer reaction times (3–6 h) for full phenolic reduction However, this can be optimized through enzyme immobilization or by combining laccase with mechanical clarification methods^60^. In the current study, enzyme profiling of the crude extract revealed prominent laccase and α-amylase activities, while pectinase and xylanase activities were minimal, and cellulase activity was undetectable. The reduction in turbidity and phenolic content is therefore attributed primarily to laccase, while viscosity changes may have been partially influenced by amylase.

The physicochemical parameters of fresh apple juice as well as laccase treated samples are presented in Table 1. The results indicated that juice yield was low in fresh apple juice (71.59%) compared to laccase treated sample (87.34%). The obtained results are in agreement with Pilnik and Vorange^61^ who referred to the importance of using enzymes in fruit processing to get high yield of juice, filtration improvement and obtain clear juices with high quality for concentration process. The increase in juice yield is mostly correlated with the increase in soluble sugar in juice. During fruit enzymation, the middle lamella as well as pectin of cell wall of the product are degraded and transformed to soluble material like neutral sugars and acids^62^. Apple juice subjected to enzyme treatment exhibited considerably (P < 0.05) lower pH values than the control samples, and both samples showed a considerable rise over storage (Table 1). These findings are consistent with those of Marc et al.^63^, who observed that cashew apple juice’s pH decreased after storage following heat treatment. The breakdown of pectin and the creation of galacturonic acid following enzyme treatment might be the cause of the pH rise during storage. While the pH of the enzyme-treated sample recorded 4.05 to 4.31 throughout the storage period, the pH of the untreated apple juice varied from 4.12 to 4.33.

**Table 1:** Impact of laccase treatment on physicochemical characteristics of apple juice.

Characteristics	Control	Enzyme-treated juice
Juice yield	71.59 ± 0.28	87.34 ± 0.19
Time (day)	pH	
Zero	4.12 ± 0.02^b^	4.05 ± 0.07^c^
2	4.16 ± 0.06^b^	4.12 ± 0.02^d^
4	4.23 ± 0.03^d^	4.19 ± 0.06^e^
7	4.28 ± 0.05^a^	4.25 ± 0.08^a^
10	4.30 ± 0.03^a^	4.29 ± 0.04^a^
14	4.33 ± 0.08^e^	4.31 ± 0.03^f^
Titratable acidity (TA) (%)		
Zero	0.358 ± 0.02^d^	0.438 ± 0.05e
2	0.329 ± 0.06^c^	0.431 ± 0.06^a^
4	0.308 ± 0.05^b^	0.429 ± 0.08^a^
7	0.295 ± 0.08^b^	0.425 ± 0.04^b^
10	0.281 ± 0.04^a^	0.416 ± 0.03^c^
14	0.276 ± 0.07^a^	0.397 ± 0.09^d^
Total soluble solids (TSS)		
Zero	9.58 ± 0.25^a^	11.23 ± 0.23^a^
2	9.62 ± 0.16^a^	11.65 ± 0.15^b^
4	10.07 ± 0.13^b^	11.92 ± 0.16^c^
7	10.63 ± 0.18^c^	12.08 ± 0.19^d^
10	10.82 ± 0.14^d^	12.64 ± 0.17^b^
14	11.05 ± 0.12^e^	12.93 ± 0.25^e^
Apparent viscosity (cp)		
Zero	2.95 ± 0.12^c^	1.87 ± 0.08^a^
2	2.83 ± 0.16^a^	1.86 ± 0.09^a^
4	2.80 ± 0.19^a^	1.86 ± 0.06^a^
7	2.79 ± 0.14^a^	1.85 ± 0.05^b^
10	2.78 ± 0.13^b^	1.85 ± 0.04^b^
14	2.78 ± 0.18^b^	1.84 ± 0.03^b^
Browning index (BI)		
Zero	0.208 ± 0.02^a^	0.162 ± 0.06^a^
2	0.259 ± 0.05^b^	0.169 ± 0.08^a^
4	0.347 ± 0.06^c^	0.173 ± 0.09^c^
7	0.412 ± 0.09^d^	0.182 ± 0.05^d^
10	0.439 ± 0.08^e^	0.194 ± 0.04^b^
14	0.452 ± 0.04^f^	0.197 ± 0.03^b^

Values are expressed as mean ± SD, The same letters within the same column are not significant (*P* ≤ 0.05) for each characteristic.

As the storage time extended, total acidity (TA) of both untreated and laccase treated samples revealed a downward trend (Table 1). This reduction in acidity correlated with the organic acids being converted into carbohydrates or enzyme effects as well as oxidizing during juice storage^64^. The recorded TA for control apple juice and enzyme treated sample are less than value for other fruit juice strawberry 0.9%, plum 2.2%, golden berry is 1.0%, apricot 2.4% and orange 0.8% but were similar to other fruit juice TA such as pear 0.3%^62^. These differences in fresh apple juice without laccase treatment may be due to several factors such as growing environments, genetic, degree of maturity and processing methods^65^. The reduction in TA for control as well as laccase apple juice treated sample in parallel with Singh et al.^66^ who reported a reduction in TA of Kinnow juice during storage for 74 days. On the other hand, Oszmiański and Wojdyło^67^ did not found any significant change in apple juice acidity when they study the effect of traditional method of clarification.

The results cited in Table 1 showed that TSS values of apple juice treated by laccase were higher than those recorded with untreated samples. The obtained results are in agreement with published study^68^. Additionally, Table 1 demonstrated TSS values of control apple juice to be 9.58 at zero time and reached 11.05 at the end of storage period. These values were less than the TSS levels in fresh apple juice (11.2 and 11.80) reported by Hameed et al.^69^ respectively. A significant increase was observed for TSS in laccase apple juice treated sample which had 11.23 at zero time and recorded 12.93 after two weeks of storage at 4 °C (Table 1). Similar data for TSS increase during storage was found in mandarin, lemon and sweet orange. Increase of TSS during storage may be due to hydrolysis of polysaccharides (starch) into monosaccharides (sugars), increase in concentration of juice due to dehydration and degradation of pectic substances of juice in soluble solids. The increase in TSS may be due to the increment of soluble sugars, which may come from the conversion of pectin or cellulose by pectinolytic or cellulase respectively to produce soluble sugars or hydrolysis of starch into sugars led to increase in TSS as reported by Hameed et al.^69^. Laccase impact on apple juice characteristics may be detected visually as the treated apple juice using laccase was more fluid and less viscous than untreated apple juice. This observation was noted in Table 1 as the viscosity of the enzyme-treated sample was 1.87 cp, whereas the viscosity of the control sample was 2.95 cp. This significant reduction in viscosity may be due to that pectinaceous substances exhibited a higher water holding capacity and make a cohesive network structure.

The effect of laccase on browning index (BI) of apple juice was studied by following the absorbance at 420 nm using spectrophotometer and the obtained results were cited in (Table 1). A gradual increase in BI was noticed during storage in both treated and untreated apple juice samples. The BI expressed about the purity of brown color and can be used as an important indicator for both enzymatic and non-enzymatic browning reactions^70^. A significant (*P* < 0.05) increase in absorbance was observed during storage. At the end of storage A_420_ was increased by about 217% and 121% in control and enzyme treated apple juice samples, respectively (Table 1). The results showed that enzyme treatment could be used as a useful method to reduce the browning by about 56%. Our finding confirmed by Bezerra et al.^19^ who clarified apple juice using laccase-glutaraldehyde-coconut fibres , achieving a 61% lightening of the original color.

### Changes in total phenolic compounds and antioxidant activity of apple juice with laccase treatment

Although the polyphenols in fruit juice have positive nutritional and therapeutic effects on humans, they also contribute to the formation of sediments or haze because of their reaction with proteins causing turbidity, altering the product’s flavor, aroma and shortening its shelf life^71^. The objective of the present study was to apply laccase from *Pleurotus ostreatus* NRC 620 to reduce the level of phenolic compounds in apple juice in a safe way. The results cited in Table 1 indicated a significant decrease in the total phenolic content of apple juice after treatment with laccase before storage in a refrigerator at 4 °C. Also, a decrease in total phenolic content was detected during storage in both studied samples (Table 1). According to Sandri et al.^72^, apple juice treated with enzymes retains its antioxidant activity and phenolic content. However, findings reported by Lettera et al.^73^ showed that treatment of orange juice with fungal laccase might reduce its phenol level by up to 45%.

Phenolic compounds were confirmed to act as free radical scavengers, reducing agents, singlet oxygen quenchers, and hydrogen atom transfer as well as electron donors to free radicals which make them potent antioxidants^74^. So, antioxidant activity based on DPPH and FRAP methods was used to evaluate the effect of laccase on the antioxidant activity of apple juice during storage for 14 days in a refrigerator (Table 2). In both methods, there were increases in antioxidant activity during storage which may be due to an increase of free phenolic compounds or the formation of Maillard reaction products (MRPs) which might be responsible for the improvement of antioxidant activity^75^. The formation of brown pigments (melanoidins) via non-enzymatic browning reactions including ascorbic acid degradation, Maillard reaction sugars acid-catalyzed degradation. Ascorbic acid degradation intermediated and sugar degradation products like carbonyl compounds can react with amino acids and participate in Maillard-associated reactions^76^. While the browning of fruit and vegetables during storage has been studied intensively, the understanding of these reactions is still limited^77^. Apple juice that was treated with laccase enzyme exhibited lower significant antioxidant activity based on the DPPH method in comparison with FRAP assay (Table 2), as well a significant increase in all samples with prolonged storage time was observed. In this study, two different assays were used to determine the antioxidant activity as they work under different principles. DPPH assay measures the ability to scavenge free radicals, and the FRAP assay measures the capacity to reduce ferric ions. Therefore, it is recommended to use more than an assay to determine the antioxidant activity to have a better elucidation of the antioxidant activity of the investigated sample^78^.

**Table 2:** Influence of laccase treatment on total phenolic content and antioxidant activity of apple juice.

Characteristics	Control	Enzyme-treated juice
Juice yield	71.59 ± 0.28	87.34 ± 0.19
Total phenolic content (mg/mL)		
Zero	0.865 ± 0.19^a^	0.827 ± 0.12^b^
2	0.861 ± 0.22^a^	0.816 ± 0.15^c^
4	0.823 ± 0.13^c^	0.807 ± 0.16^a^
7	0.819 ± 0.15^b^	0.802 ± 0.18^a^
10	0.813 ± 0.17^b^	0.799 ± 0.09^d^
14	0.805 ± 0.14^d^	0.786 ± 0.12^e^
Antioxidant activity		
DPPH (µM Trolox/mL)		
Zero	536.7 ± 0.34^a^	425.7 ± 0.18^b^
2	549.2 ± 0.25^b^	446.3 ± 0.29^c^
4	553.8 ± 0.19^c^	458.2 ± 0.31^a^
7	596.4 ± 0.37^d^	461.9 ± 0.17^a^
10	612.6 ± 0.28^e^	472.5 ± 0.12^d^
14	645.3 ± 0.16^f^	486.9 ± 0.28^d^
FRAP (µM Trolox/mL)		
Zero	634.5 ± 0.34^c^	612.6 ± 0.16^a^
2	651.2 ± 0.26^d^	634.5 ± 0.24^b^
4	659.8 ± 0.37^a^	651.7 ± 0.18^c^
7	662.3 ± 0.45^a^	662.8 ± 0.12^d^
10	678.4 ± 0.13^b^	673.4 ± 0.19^e^
14	681.5 ± 0.18^b^	682.9 ± 0.14^f^

Values are expressed as mean ± SD, the same letters within the same column are not significant (*P* ≤ 0.05) for each characteristic.

One of the study’s main conclusions is that the laccase from *Pleurotus ostreatus* NRC 620 functions best at 70 °C and pH 3.0. *P. ostreatus* NRC 620 exhibits greater thermal stability and a more acidic pH preference than other fungal laccases frequently used in juice clarification, such as *Trametes versicolor* and *Ganoderma lucidum*, which normally have optimum temperatures in the range of 50–60 °C and pH values between 3.5 and 5.0. This difference might lead to improved juice clarifying efficiency, especially for acidic fruit juices where stability at low pH levels is advantageous. Compared to other fungal laccases that have been investigated, *P. ostreatus* NRC 620 is unusual in that it can operate well in more severe environments. The higher temperature optimum points to possible benefits for industry, such as faster reaction times and less microbial contamination. Juice clarifying procedures can benefit from its lower pH preference, which also fits well with many fruit juices’ acidic quality. These results merit more investigation in large-scale applications, positioning *P. ostreatus* NRC 620 as a viable substitute for conventional fungal laccase sources. Following comparison with previous studies, we found that the ideal temperature was 60 °C, while the ideal pH was 3.0. After 80 min at 60°C, the *Ganoderma lucidum* laccase showed 46% residual activity^79^. According to Kurniawati and Nicell^80^, the *Trametes versicolor* enzyme demonstrated exceptional or moderate stability at 25 °C at pH values between 5.0, and 8.0, in addition to its stability at temperatures from 10 to 30 °C at pH 6.0. In our investigation, *Pleurotus ostreatus*'s optimal pH and temperature for enzyme activity were found to be 3.0 and 70 °C, respectively. After two hours of incubation at 40 and 50 °C, the enzyme preserved 68.33 and 59.61% of its activity. Moreover, the *Pleurotus ostreatus* NRC 620 laccase enzyme was most active throughout a wide temperature range from 50 °C to 80 °C to almost reach its maximal activity (69–98%), with its maximum activity occurring at 70 °C.

## Conclusion

In conclusion, the laccase enzyme from *Pleurotus ostreatus* NRC620, produced under static conditions, demonstrated optimal activity and stability across a range of pH and temperature conditions, highlighting its robustness compared to other enzyme sources. Supplementation with 10 mM MgSO₄ and CuSO₄ enhanced activity by approximately 21% and 35%, respectively. In apple juice treatments, the enzyme led to reductions in pH and viscosity, with only slight degradation of phenolic content during storage.

These findings support the potential application of this laccase in food processing, particularly in beverage clarification. By specifically targeting phenolic compounds, laccase not only reduces haze formation and improves clarity but also operates under mild conditions, preserving juice quality. Unlike conventional agents such as gelatin, bentonite, and silica gel, which can generate waste and remove desirable flavors, laccase offers a more sustainable and consumer-friendly alternative. Furthermore, compared to other enzymes and filtration methods, laccase provides a targeted, cost-effective solution without compromising product integrity.

## Data Availability

All data supporting the findings of this study are available on paper.
